# Analysis of functional redundancies within the *Arabidopsis* TCP transcription factor family

**DOI:** 10.1093/jxb/ert337

**Published:** 2013-10-15

**Authors:** Selahattin Danisman, Aalt D. J. van Dijk, Andrea Bimbo, Froukje van der Wal, Lars Hennig, Stefan de Folter, Gerco C. Angenent, Richard G. H. Immink

**Affiliations:** ^1^Plant Research International, Bioscience, Droevendaalsesteeg 1, 6708 PB Wageningen, The Netherlands; ^2^Swedish University of Agricultural Sciences, Uppsala BioCenter, Department of Plant Biology and Forest Genetics, PO Box 7080, SE 75007, Uppsala, Sweden; ^3^Laboratory of Molecular Biology, Wageningen University, 6700 ET, Wageningen, The Netherlands; ^4^Consortium for Improving Plant Yield (CIPY), PO Box 98, 6700 AB, Wageningen, The Netherlands

**Keywords:** Bioinformatics, gene regulation, leaf development, redundancy, senescence, TCP transcription factor.

## Abstract

Analyses of the functions of TEOSINTE-LIKE1, CYCLOIDEA, and PROLIFERATING CELL FACTOR1 (TCP) transcription factors have been hampered by functional redundancy between its individual members. In general, putative functionally redundant genes are predicted based on sequence similarity and confirmed by genetic analysis. In the TCP family, however, identification is impeded by relatively low overall sequence similarity. In a search for functionally redundant TCP pairs that control *Arabidopsis* leaf development, this work performed an integrative bioinformatics analysis, combining protein sequence similarities, gene expression data, and results of pair-wise protein–protein interaction studies for the 24 members of the *Arabidopsis* TCP transcription factor family. For this, the work completed any lacking gene expression and protein–protein interaction data experimentally and then performed a comprehensive prediction of potential functional redundant TCP pairs. Subsequently, redundant functions could be confirmed for selected predicted TCP pairs by genetic and molecular analyses. It is demonstrated that the previously uncharacterized class I *TCP19* gene plays a role in the control of leaf senescence in a redundant fashion with *TCP20*. Altogether, this work shows the power of combining classical genetic and molecular approaches with bioinformatics predictions to unravel functional redundancies in the TCP transcription factor family.

## Introduction

TEOSINTE-LIKE1, CYCLOIDEA, and PROLIFERAT ING CELL FACTOR1 (TCP) transcription factors constitute a small family of plant-specific transcription factors whose members share functions in plant development (for a review, see Martín-Trillo and Cubas, 2010). The *Arabidopsis thaliana* genome encodes for 24 TCP transcription factors, which are divided into class I and class II TCPs based on sequence similarities (Cubas *et al.*, 1999; Kosugi and Ohashi, 2002). All TCP transcription factors share the TCP domain, a 59-amino-acid-long, non-canonical basic helix–loop–helix domain responsible for nuclear targeting, DNA binding, and mediating protein–protein interactions (Cubas *et al.*, 1999; Kosugi and Ohashi, 2002). Apart from this domain, TCP protein sequences are, in general, highly variable. Analysis of single *tcp* knockout mutants in *Arabidopsis* resulted in only a few distinct and mainly subtle mutant phenotypes (Takeda *et al.*, 2006; Schommer *et al.*, 2008; Tatematsu *et al.*, 2008; Danisman *et al.*, 2012). The majority of known *Arabidopsis tcp* mutant phenotypes are the result of double or multiple knockouts. In the *JAGGED AND WAVY* (*JAW*-D) mutant for instance, overexpression of the microRNA *miR319a* leads to the knockdown of five class II *TCPs* (below referred to as *jaw-TCPs*): *TCP2*, *TCP3*, *TCP4*, *TCP10*, and *TCP24*. *Jaw*-D plants exhibit several phenotypic defects, including highly serrated leaves, altered petal development, and delayed leaf senescence (Palatnik *et al.*, 2003; Schommer *et al.*, 2008; Nag *et al.*, 2009). Part of these phenotypes could be explained upon closer examination of genes that act downstream of TCP4 (Schommer *et al.*, 2008), although a list of direct target genes is missing for this regulatory protein. Overexpression of microRNA-insensitive *TCP4* leads to developmental arrest in an early seedling stage, characterized in part by a lack of the shoot apical meristem (SAM) (Palatnik *et al.*, 2003). The *tcp4* single-knockout phenotype shows only a mild leaf serration phenotype, which can be enhanced by introducing knockouts of the other *jaw-TCPs* (Schommer *et al.*, 2008). The degree of phenotype alterations varies and depends on which *tcp* mutant is being combined with *tcp4* plants, suggesting that the five *jaw-*TCPs share only partially redundant functions. In the class I TCP clade, only a few phenotypes are known, the most recently described are the *tcp14tcp15* (Kieffer *et al.*, 2011; Steiner *et al.*, 2012) and the *tcp9tcp20* double mutants (Danisman *et al.*, 2012). Considering that there are 14 class I TCPs, for most of which no phenotype is known, the *Arabidopsis* TCP transcription factor family is far from fully explored and there is still potential to unravel functions based on the combination of different knockout mutants.

The high degree of redundancy in the TCP transcription factor family constitutes a problem for functional analyses of members of this family. Full genetic redundancy is evolutionary instable (Thomas, 1993) because the duplication of a gene lowers the selective pressure on both the new copy and the original gene (Hughes, 1994). This means in general that TCPs can be expected to show subfunctionalization rather than full genetic redundancy: they share common functions but have also distinct roles and expression patterns (Briggs *et al.*, 2006). Hence, additional functional information is essential for the identification of redundant TCP pairs for a specific biological process. This information can be achieved by integrating sequence information with gene expression data and information about features of the encoded proteins.

This work combined bioinformatics and experimental approaches to identify TCP transcription factor pairs that share functionality in *Arabidopsis* leaf development and determined TCP protein pairs that probably share functions in *Arabidopsis* leaf development. Both known and unknown TCP pairs were identified and functional redundancies for exemplary cases were validated using classical genetics and molecular approaches.

## Materials and methods

### Plant material

Seeds from the original *jaw*-D mutant (Palatnik *et al.*, 2003) were used. For *TCP8*, *TCP19*, *TCP20*, and *TCP22*, T-DNA insertion lines were obtained from the Nottingham *Arabidopsis* Stock Centre (*tcp8*, SAIL_656_F11; *tcp19*, SALK_024434.47.85.x; *tcp20*, SALK_016203.45.25.x; *tcp22*, SALK_045755.56.00.x), and homozygous insertion mutants were selected based on gene-specific PCR experiments.

### Plant growth conditions

Plant material for the leaf expression analysis and the senescence assays was grown under long-day conditions (16/8 light/dark cycle, 21 °C) on rockwool and received 1g l^–1^ Hyponex plant food solution twice per week. Plants for dexamethasone (DEX) induction experiments were grown on half-strength Murashige and Skoog (MS) salts supplemented with 8g l^–1^ agar).

### Constructs

For the glucocorticoid induction experiments, this work created a microRNA-insensitive version of *TCP10* (*TCP10m*) by site-directed mutagenesis and cloned it into a glucocorticoid receptor (*GR*) destination vector. The *TCP10* miRNA target site was mutated in the same way as it was done previously for *TCP4* (Palatnik *et al.*, 2003). Primers used are given in Supplementary Table S6. These primers allow mutation of the *miR319a* binding site without changing the expressed protein’s amino acid sequence. *TCP10m* was cloned into the GATEWAY-compatible pCR8/GW/TOPO vector (Invitrogen). It was then placed behind the CaMV35S promoter in a *GR* destination vector (pARC146; Danisman *et al.*, 2012) via an LR reaction.

### Transformation of *Arabidopsis*

Wild-type *A. thaliana* (accession Columbia-0) plants were grown on soil until the primary inflorescences emerged, which were cut to promote growth of secondary inflorescences and to increase the number of floral buds. The binary *TCP10m-GR* construct was transformed into *Agrobacterium tumefaciens* strain C58C1-PMP90. Transformation of plants was conducted by floral dip (Clough and Bent, 1998). After transformation, plants were kept in a growth chamber until seed set. The T1 seeds were then selected on germination medium containing 30 µg ml^–1^ kanamycin for 2 weeks, after which rooting green T1 seedlings were transferred to soil and grown until seed set. The following T2 generation was checked for expression of the transgene by reverse-transcription PCR.

### RNA isolation and qRT-PCR

RNA was extracted with lithium chloride/phenol/chloroform (Verwoerd *et al.*, 1989). DNase (Invitrogen) treatment was stopped with 1 µl of a 20-mM EDTA solution and 10min incubation at 65 °C, RNA concentration was measured, and 500ng RNA was used to perform cDNA synthesis. The cDNA was diluted 10 times and used for quantitative real-time PCR (qRT-PCR) using the SYBR green mix from BioRad. The SAND family gene, *AT2G28390*, which was determined as ‘superior reference gene’ for developmental studies (Czechowski *et al.*, 2005), was used as reference gene for the analyses. The qRT-PCR data was analysed using the ΔCT method (Livak and Schmittgen, 2008). Expression was given in relation to the reference gene only, without normalizing to a specific time point. The error bars depict the biological variation between three independent biological replicates (standard error, SE). The primers used in the transcript analyses are given in Supplementary Table S6.

### Yeast two-hybrid analysis

Protein–protein interactions between TCP proteins were analysed in a matrix-based yeast two-hybrid (Y2H) GAL4 assay (de Folter *et al.*, 2005). Bait vectors were transformed into yeast strain PJ69-4α; prey vectors were transformed into yeast strain PJ69-4a (James *et al.*, 1996). The individual transformants were grown in liquid synthetic dropout (SD) medium lacking Leu and Trp, respectively. These overnight cultures were mated by spotting 5 µl liquid culture of the individual yeast cultures on top of each other on SD-complete plates, containing all essential amino acids. After overnight incubation, yeast was transferred by a 96-pins replicator to freshly prepared SD plates lacking both Leu and Trp, selecting for diploid yeast containing the two plasmids. In a last step, the mated yeast clones were transferred on SD–Leu–Trp–Ade or SD–Leu–Trp–His medium, supplemented with 5 and 10mM 3-amino-1,2,4-triazole, respectively. Growth of yeast, and hence protein–protein interaction events, was scored after 5 days at 30 °C. Because of high auto-activation capacity of several TCPs, not all combinations could be analysed reciprocally. Auto-activation capacity was determined beforehand for the baits by testing for growth of the single pBDGAL4-TCP transformants on selective SD medium for the His and Ade markers. TCP1, 2, 4, 10, 12, 18, 20, and 24 exhibited auto-activation when expressed from the GAL4 BD vector and matings with these particular TCP-BD constructs were excluded from the matrix-based Y2H analysis. Every combination was analysed 18 times (six replicates and three different selection media). In the end, only pairs that reproducibly scored positive with at least two different selection markers were taken as true protein–protein interactions.

### Dexamethasone induction experiments

Plants were treated with DEX continuously to see phenotypic effects of *TCP10m* overexpression, whereas DEX treatment was given only transiently to 5-day-old seedlings in order to find possible target genes in the induced transcriptome.

Continuous DEX treatment was achieved by including 10 µM DEX into the germination medium. Transient DEX induction experiments were conducted using a transfer system facilitated by nylon meshes. Per plate, 30–50 seeds were sown on top of a 200-µm nylon mesh that was placed onto germination medium with 6g l^–1^ instead of 8g l^–1^ agar. Because the plants were grown on nylon meshes on top of low-concentrated agar, they could be transferred into induction media quickly and without severely damaging the roots. The induction medium consisted of half-strength MS, 1% (w/v) sugar, 10 µM DEX, and 10 µM cycloheximide (CYC). Samples for RNA isolation were harvested immediately before and 4h after start of the treatment.

### Senescence assays

Plants were cultivated for 24 days, and the fifth and sixth leaves were detached and placed in a randomized way into 24-well plates, floating on milliQ water. These plates were incubated in the dark for 4 days. Photographs were taken and leaves were classified, based on leaf colour into four classes, with class I representing healthy green (non-senescing) leaves and class IV representing completely yellow and senescent leaves. Distributions over the four classes were compared between the mutant lines using a chi-squared test.

### Microarray analysis

Transcript profiling starting with 1 µg of DNA-free RNA was performed using Affymetrix *Arabidopsis* AGRONOMICS1 tiling microarrays (Affymetrix, Santa Clara, CA). Labelling of samples, hybridizations, and measurements were performed as described by Rehrauer *et al.* (2010). Signal values were derived using the RMA algorithm implemented in the statistical language R (R Development Core Team, 2010) using probe sets comprising exonic probes based on the TAIR10 genome annotation. For details of probe set definition and low-level data analysis, see Rehrauer *et al.* (2010). Differentially expressed genes were selected using the RankProduct algorithm (Breitling *et al.*, 2004). Genes were considered as differentially expressed if *P* < 0.05. The microarray data is available on ArrayExpress (accession number: E-TABM-1191). The BiNGO 2.44 plugin for Cytoscape (Maere *et al.*, 2005) was used with standard settings (Benjamini-Hochberg FDR, significance level of 0.05) to search for overrepresented gene ontology terms.

### Computational analysis

*A. thaliana* protein sequences were obtained from TAIR10 and their phylogeny was constructed using PhyML (Guindon and Gascuel, 2003) with the JTT substitution model, a distance-based tree as starting tree and maximum likelihood estimation for the gamma distribution parameter. Trees were visualized using the R-package APE (Paradis *et al.*, 2004).

To generate trees based on the Y2H or gene expression datasets, the information in those datasets was first converted to distances between pairs of TCPs. Based on the Y2H dataset, the distance for each protein pair was calculated as the number of proteins that were interacting with only one out of this pair. This is equivalent to encoding the interaction pattern of each protein as a binary vector with 1 indicating interaction and 0 indicating non-interaction, and then calculating the distance between two proteins by subtracting two vectors and using the squared length of the resulting vector. The distance matrix obtained by calculating this distance for each pair of proteins was subsequently scaled such that the maximum value was 1.0 and the minimum value 0.0 by applying a linear transformation: *d*_new_=(*d*_old_ – *d*_min_)/(*d*_max_ – *d*_min_), where *d*_old_ and *d*_new_ indicate the values of the distance before and after transformation, respectively; *d*_max_ and *d*_min_ indicate the maximum and minimum distance before scaling. The scaling does not change the relative ordering of pairs of proteins but makes distances more comparable when comparing different datasets. For the gene expression datasets [AtGenExpress (Schmid *et al.*, 2005); and own data for the *TCP* genes], the distance was calculated by summing the absolute value of the difference between expression in each tissue or condition, followed by the same scaling as described above for the Y2H dataset. The way in which the distance calculation is performed for the gene expression data is completely equivalent to the calculation for the Y2H data. An alternative way to calculate the distance would be to take the square root of the sum of squares of the differences between expression in each tissue or condition; this was tested as well and found that it gives virtually indistinguishable results (Pearson correlation coefficient between the two sets of distances is ~0.99, ranking of the pairs is very similar).

For comparison of these interaction- or expression-based distances with protein sequence-based distances, a sequence-based distance matrix was obtained using ClustalW (Thompson *et al.*, 2002). The similarity between those sets of distances was characterized by the Pearson correlation coefficient. For the Y2H and expression datasets, trees were obtained based on the distance matrices using the neighbour-joining algorithm as implemented in the R-package APE (Paradis *et al.*, 2004).

To determine TCP proteins with a high potential for functional redundancy the pairs were ranked based on their distances in protein sequence, gene expression and protein–protein interaction patterns. Subsequently, these ranks were added. For simplicity, the gene expression sets were treated as being protein expression sets for calculating the ranks. In principle, one could use different weights on the ranks obtained from the different datasets, but without a large amount of training data to obtain values for such weights, each dataset was concurrently and similarly treated (all weights of 1).

To assess robustness of the obtained ranking, bootstrapping was applied as follows. For each of the different datasets, a number of resamples of the dataset (and of equal size to the observed dataset) were obtained by random sampling with replacement from the original dataset. Distances were calculated for each of those resamples, and the resulting rankings of pairs of TCPs were used in the integrative analysis to calculate the rank sum for each pair of TCPs.

## Results

### Data integration for identifying potential functionally redundant TCP pairs

**Fig. 1.**
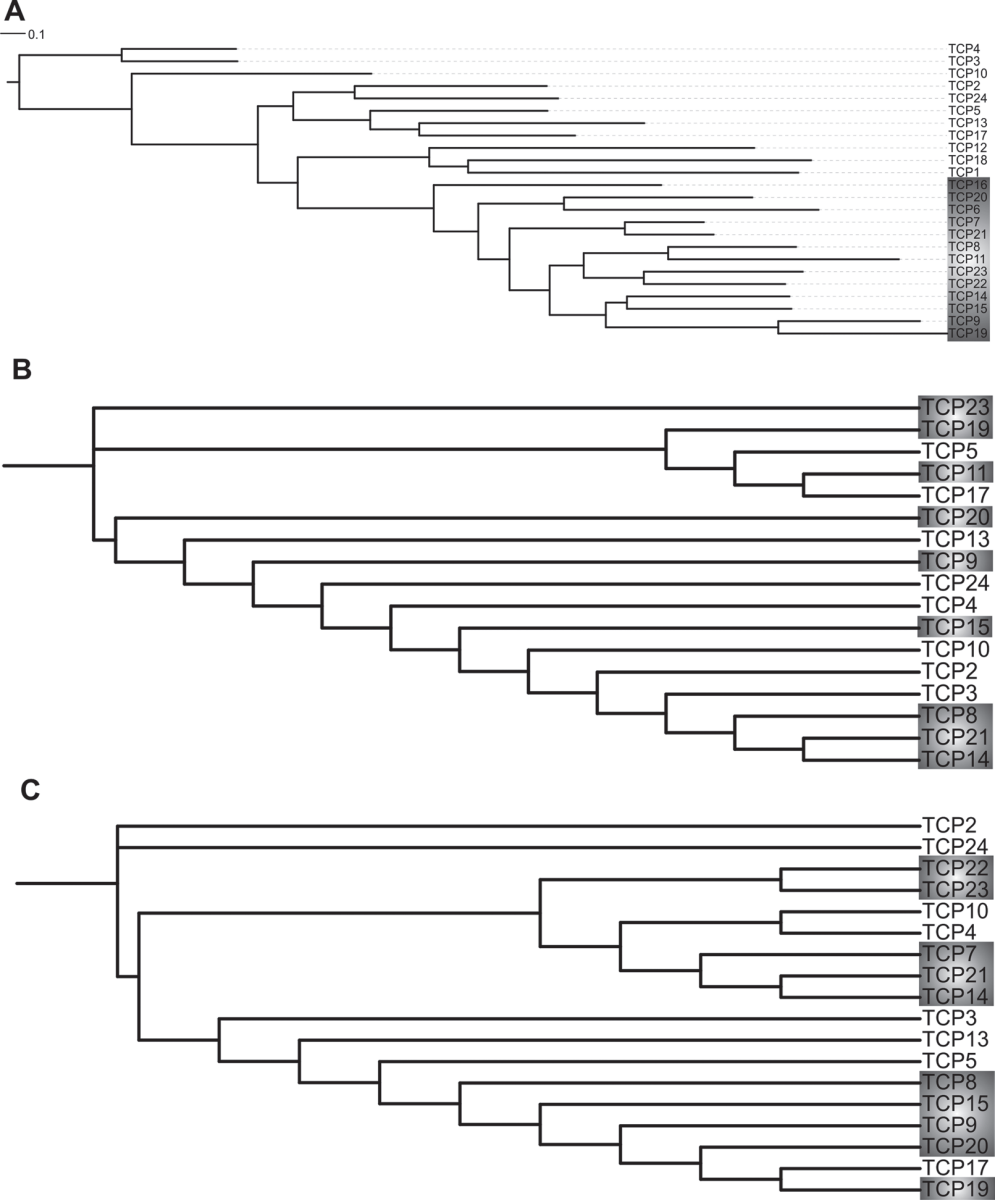
Relationships between TCP transcription factors. Relationship is based on (A) protein sequence, (B) AtGenExpress data, and (C) quantitative real-time PCR data on leaf development. The phylogenetic tree for TCP protein sequences was generated using PhyML. Trees representing expression data were generated by first converting expression patterns to distances between pairs of genes and then applying the neighbour-joining algorithm. Expression data for (B) were from the AtGenExpress microarray expression compendium by Schmid *et al.* 2005; expression data for (C) are from this study. class I TCPs are marked in grey.

**Fig. 2.**
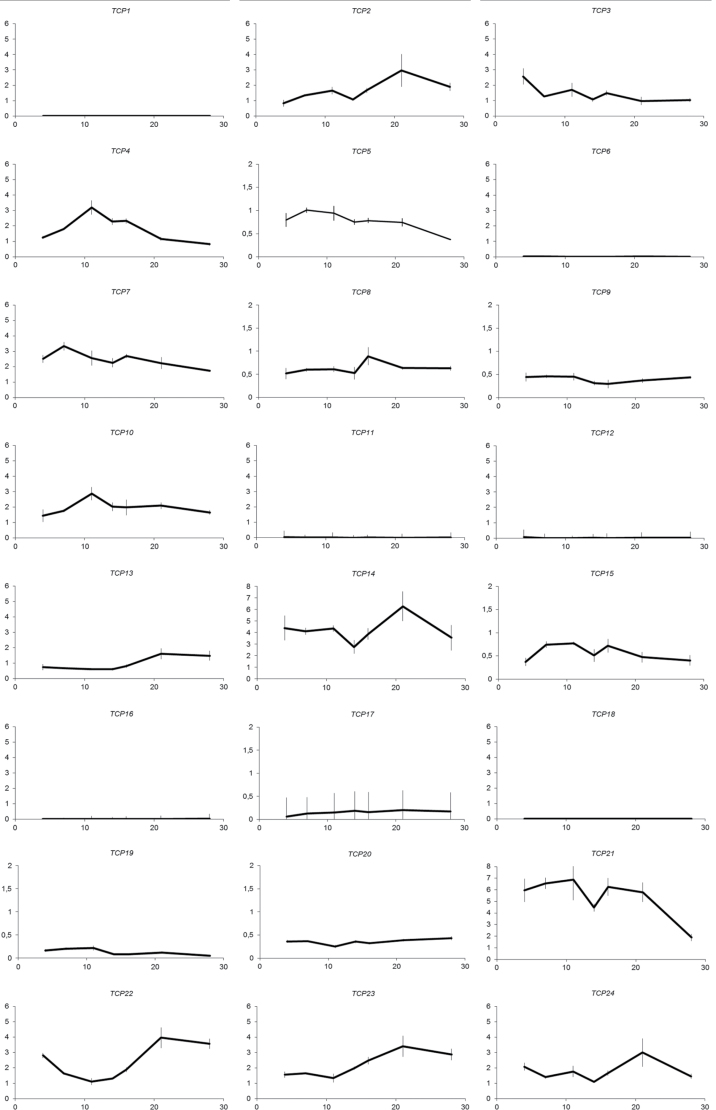
Expression analysis of all 24 *Arabidopsis TCP* transcription factor genes during leaf development. Quantitative real-time PCR was performed on seedlings harvested at days 4 and 7 after germination and on the first rosette leaf harvested at days 11, 14, 16, 21, and 28 after germination. Analysis was done in triplicate and bars indicate SE. X-axis, time in days; Y-axis, normalized expression.

This work started with the hypothesis that comparing the behaviour of members of a gene family in various datasets, and identifying those that behave in the most similar way based on this information, would yield a list of gene combinations that are likely to share common molecular and biological functions. Because whole-genome and chromosomal segment duplications are an important source for additional copies of genes in plant genomes (Flagel and Wendel, 2009), the prediction focused on pairs of *TCP* genes. For this purpose, information on DNA and protein sequences and expression patterns can be obtained from publicly available resources. However, further in-depth bioinformatics analysis using comprehensive datasets may increase the predictive power of the data integration. Therefore, a data analysis pipeline was built, in which phylogenetic relationships, gene expression patterns, and information about protein–protein interaction capacity are integrated. Initially, a phylogenetic tree was built based on publicly available protein sequence data for all 24 *Arabidopsis* TCP transcription factors. Similar to previously described phylogenetic trees (Cubas *et al.*, 1999; Aguilar-Martinez *et al.*, 2007), the resulting phylogenetic tree divides the TCP family into two distinct classes, the class I and class II TCP proteins (Fig. 1A). In a next step, differences in *TCP* expression during *Arabidopsis* development were analysed based on publicly available microarray data from AtGenExpress (Schmid *et al.*, 2005). Some of the *TCP* genes did not show significant expression for any of the analysed tissues: these were *TCP1*, *TCP6*, *TCP7*, *TCP12/BRC2*, *TCP16*, *TCP18/BRC1*, and *TCP22* (Supplementary Table S1). As this work were specifically interested in functional redundancies of *TCP* genes in leaf development, the AtGenExpress data were extended by analysing expression of all 24 *TCP* genes by qRT-PCR in a leaf developmental series. In this experiment, all aboveground parts of wild-type *Arabidopis* Col-0 seedlings were harvested at days 4 and 7 after germination, and the first rosette leaf was harvested at days 11, 14, 16, 21, and 28 after germination. Per time point, three biological replicates consisting of 30 plants were harvested. Also here, for some *TCP*s no expression was detected (*TCP1*, *TCP6*, *TCP11*, *TCP12/BRC2*, *TCP15*, *TCP18/BRC1*), indicating that these are not or only very weakly expressed in leaves (Fig. 2). Subsequently, expression-based distances between pairs of *TCP* genes were calculated for the AtGenExpress dataset and the newly generated expression data (Fig. 1B and C). The calculated distances were correlated between the two expression datasets; however, as expected the distance scores do not overlap perfectly (Supplementary Fig. S1). Therefore, the two expression-based scores were integrated separately into the bioinformatics analysis for the prediction of TCP transcription factor pairs that share functions in leaf development.

Some *TCP* gene pairs show a high expression correlation, but they would not have been denominated as closest related *TCP* genes based on similarity of the encoded protein sequences. For example, TCP14 and TCP21 are class I TCP proteins that do not cluster in the same subclade based on protein sequence comparison (Fig. 1A), but were found to be highly co-expressed in a variety of tissues (Fig. 1B) and during leaf development (Fig. 1C) and thus potential candidates for functional redundancy based on similar gene expression patterns. Although, functional analyses are still needed to proof redundancy in this particular case, it is an example of two TCPs that would not have been considered based on protein sequence similarity solely.

### A matrix-based Y2H analysis shows class preference in TCP–TCP interactions

**Fig. 3.**
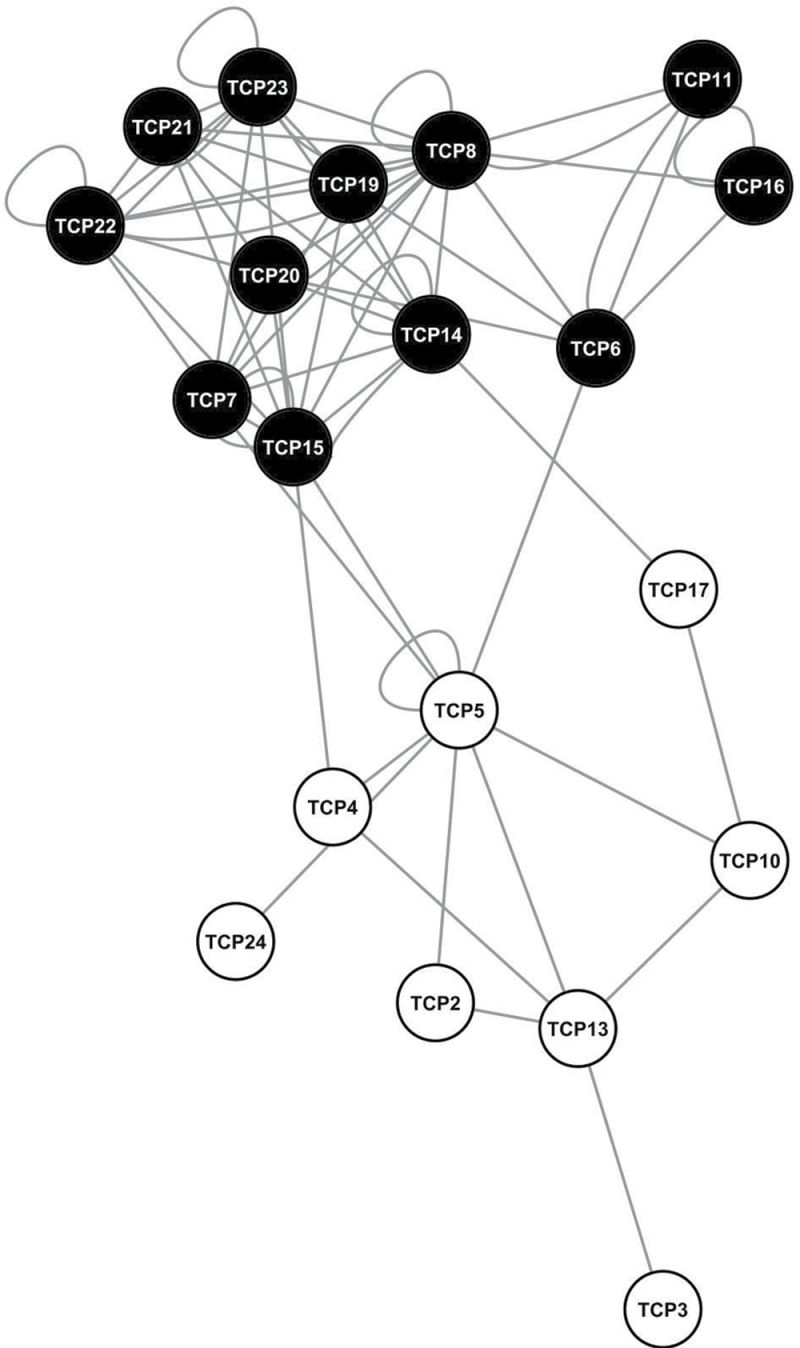
Results of the matrix-based yeast two-hybrid analysis of TCP–TCP protein–protein interactions. Cytoscape version 2.6.2 (Shannon *et al.*, 2003) was used to visualize protein–protein interactions. Nodes represent the TCPs, edges represent the protein–protein interaction between these. White nodes are class II TCPs, black nodes are class I TCPs. As the graphical layout is spring embedded, groups of nodes are placed closer to each other equivalent to the number of edges between them. The representation reveals that TCPs prefer protein–protein interactions within their own class.

TCP transcription factors are known to form dimers (Kosugi and Ohashi, 2002) and to interact with other type of proteins (Pruneda-Paz *et al.*, 2009; Giraud *et al.*, 2010). The current work investigated protein–protein interaction capabilities for all 24 *Arabidopsis* TCP transcription factors in a matrix-based Y2H analysis to obtain additional functional data for TCP transcription factors that can be implemented to improve the prediction of functional redundancy. The assay resulted in 64 detected dimer combinations: seven homodimers and 57 heterodimers (Fig. 3).

Class I TCP transcription factors prefer to interact with other class I TCP proteins, and the same holds for class II TCP proteins. Based on phylogeny, class II TCP proteins grouped into three subclades, of which members from the CYCLOIDEA/TEOSINTE BRANCHED-like subclade (TCP1, TCP12, and TCP18) did not show any interaction in the Y2H analysis. A recent study in *Gerbera* suggests that these TCP proteins homodimerize and interact specifically with each other (Tähtiharju *et al.*, 2012). However, due to auto-activation of the reporters, the homodimerization capacity for the *Arabidopsis* CYCLOIDEA/TEOSINTE BRANCHED-like proteins could not be accessed. The other two subclades of the class II TCP proteins consist of CINCINNATA-like TCP transcription factors, and they differ in the fact that the genes belonging to one subclade are targeted by *miRNA319* (*TCP2*, *TCP3*, *TCP4*, *TCP10*, *TCP24*, the so-called *jaw*-*TCP* genes), and members from the other are not (*TCP5*, *TCP13*, *TCP17*, hereafter called *TCP5*-like *TCP* genes). Interestingly, *jaw*-TCP proteins preferably form dimers with TCP5-like proteins, and vice versa. This phenomenon of preferred dimerization between members from different subclades was not detected for the class I TCP transcription factors. Remarkably, the number of potential dimerization partners per protein was higher for class I TCP proteins (average of 7.3 interaction partners in comparison to class II TCPs with an average of 2.5). It is not known whether this is of functional relevance or whether it is due to the fact that class II TCPs, in contrast to class I TCPs, exhibit more often autoactivation capacity in yeast and, hence, could not be tested for all possible combinations.

### Integrative analysis of TCP transcription factors leads to several functional redundancy predictions

In a next step, pairs of TCP proteins with a high potential for functional redundancy were determined by ranking the distances in protein sequence, gene expression, and protein–protein interaction patterns of all TCP pairs and cumulating the ranks. Due to the slight differences between the two gene expression datasets, and because integrating both expression datasets would need a correction for a bias towards expression data at the expense of sequence homology and protein–protein interaction data, the two datasets were used independently and obtained two different rankings. *TCP6* and *TCP16*, which could not be detected in either of the expression datasets, were excluded from further analysis because no score could be calculated. Nevertheless, similar functions have been reported for *TCP11* and *TCP16* (Takeda *et al.*, 2006; Viola *et al.*, 2011). Likewise, TCP proteins that exhibited no interaction in the Y2H assay were also excluded from the analysis: these were TCP1, TCP12, and TCP18. The full information on calculated redundancy scores is given in Supplementary Tables S2 and S3. Before analysing specific TCP pairs, the robustness of the ranking was tested by applying bootstrapping on the different datasets, recalculating the different ranks, and obtaining the rank sum after combining the different information sources. This bootstrapping analysis indicated that the ranking was indeed robust: compared to the rank sum obtained using the original dataset, the average Spearman rank correlation obtained was 0.98±0.02 (range 0.89–1.0). The 10 TCP pairs with the best scores (i.e. the ones with the lowest rank sum using the integrated datasets) are listed for the two independent integrative analyses in Table 1. Although there is a high correlation between the two analyses when comparing all rankings (Spearman’s rank correlation 0.94, *P* < 10^–15^), the top 10 tables for functionally redundant TCP pairs show some differences. This may be due to the strong cut off applied when only counting the best 10 out of 136 and 120 analysed TCP pairs, respectively. Also, one of the pairs obtained by the analysis using the microarray data involves TCP11, for which no expression of the encoding gene could be detected in leaves by qRT-PCR. Hence, this pair could obviously not be predicted by the analysis based on qRT-PCR data. Some TCP pairs appear in both tables: TCP19–TCP20, TCP13–TCP17, TCP4–TCP10, TCP2–TCP24, and TCP3–TCP4. A large number of these combinations were described to be functionally redundant in previous studies (Palatnik *et al.*, 2003; Efroni *et al.*, 2008), showing that the approach was solid and identified the majority of known cases. Interestingly, this approach also predicted various novel combinations, as exemplified by one of the top hits, TCP19–TCP20. This result suggests that, although sequence similarity is a strong predictor of functional redundancy, available information about expression or protein behaviour can be additive and useful.

**Table 1.** Top 10 pairs of TCP transcription factors predicted to be most likely candidates for functional redundancyThe ranks were based on an integrated analysis of protein sequence, yeast two-hybrid, and gene expression data (either AtGenExpress or quantitative real-time PCR) for leaf development.

AtGenExpress		Quantitative real-time PCR	
Rank	TCP pair	Rank	TCP pair
1	TCP13–TCP17	1	TCP22–TCP23
2	TCP19–TCP20	2	TCP19–TCP20
3	TCP2–TCP24	3	TCP2–TCP24
4	TCP3–TCP4	4	TCP4–TCP10
5	TCP5–TCP17	5	TCP13–TCP17
6	TCP4–TCP10	6	TCP3–TCP4
7	TCP20–TCP23	7	TCP8–TCP15
8	TCP17–TCP24	8	TCP15–TCP20
9	TCP15–TCP23	9	TCP7–TCP23
10	TCP11–TCP19	10	TCP5–TCP17

### Validation of partial functional redundancies within the TCP family by molecular approaches

**Fig. 4.**
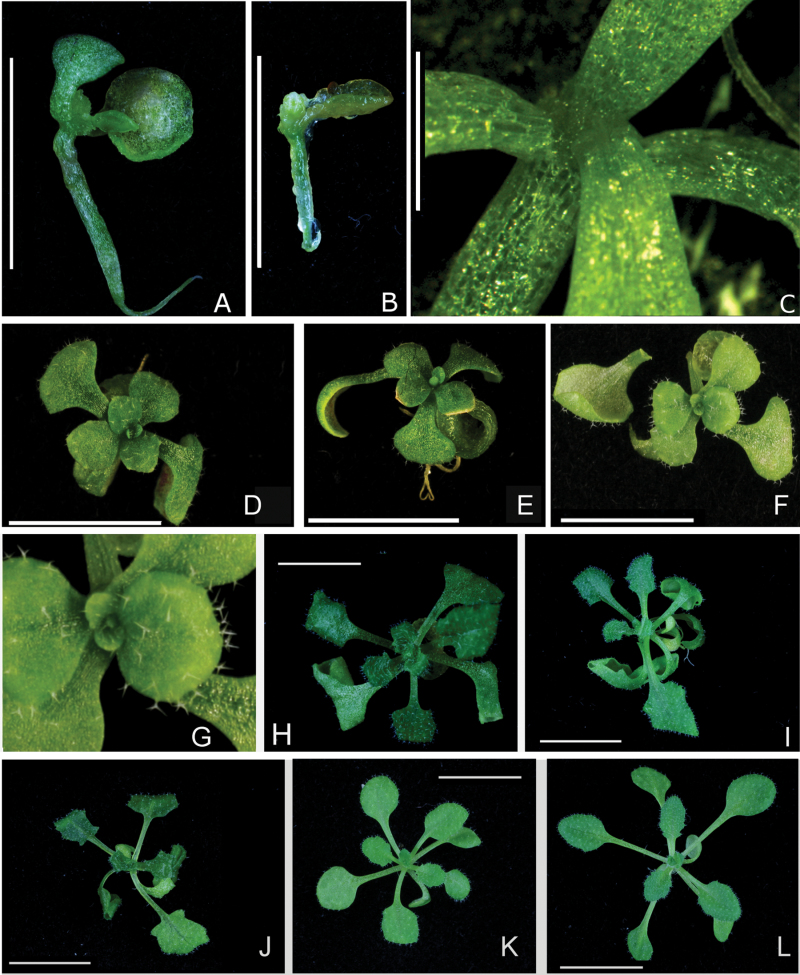
Functional complementation of the *jaw*-D phenotype by dexamethason (DEX) induction of *TCP10m-GR*. All material was grown for 3 weeks on half-strength MS medium with or without DEX treatment prior to phenotypic analyses. (A–C) Continuous induction of *jaw*-D/*TCP10m-GR* seedling by DEX leads to overcompensation; note that no new leaf primordia are formed. (D–G) When induced 6 days after germination and subsequently kept for the following 15 days on DEX, the first leaves of *jaw*-D/*TCP10m-GR* plants appeared normal, but plants remained small and eventually died. However, in these plants, various true leaf primordia were formed (G). (H) A representative 3-week-old untreated *jaw*-D/*TCP10m-GR* plant. (I) An untreated *jaw*-D control plant. (J) A *jaw*-D control plant continuously treated with DEX; note that, in contrast to the seedlings shown in (A–C), no effect of DEX is seen on the shoot apical meristem and the formation of leaf primordia. (K, L) Representative 3-week-old Col0 wild-type plants, with (K) and without (L) DEX treatment. Bars = 1cm (A, B, D–L), 0.3cm (C).

One of the best-studied and described members of the *Arabidopsis* TCP family is TCP4 and its influence on leaf development. It is known that *TCP4* and its four homologues are knocked down in the *jaw*-D genotype and that overexpression of a microRNA resistant *TCP4* leads to severe developmental defects (Palatnik *et al.*, 2003). Based on integrated analyses, *TCP10* should behave similarly upon overexpression. Hence, a *Jaw*-microRNA-insensitive *TCP10* gene (*TCP10m*) was introduced into a constitutive expression vector and tagged with a *GR* domain to allow induction of *TCP10m* at different time points during development (Aoyama and Chua, 1997). The *TCP10* mutation was introduced in the same way as described previously for *TCP4* (Palatnik *et al.*, 2003) and did not result in changes in the translated protein sequence. This *pCaMV35S*::*TCP10m*-*GR* vector was transformed into *jaw*-D plants to prevent endogenous TCP10 and other *jaw*-TCPs from competing with the introduced protein, and to test for (over-)complementation of the *jaw*-D phenotype by *TCP10m*. Continuous release of TCP10 protein into the cell nucleus by DEX treatment resulted in arrest of the SAM early during vegetative growth (Fig. 4), with the formation of only a few or no leaf primordia, phenocopying the effect of *TCP4m* (Fig. 4A–C). When the treatment was started 6 days after germination, the plants showed intermediate phenotypes: more leaves were formed, but they were smaller and non-serrated and further leaf initiation was arrested shortly after DEX induction (Fig. 4D–G). None of these observed phenotypes was seen in DEX-treated control plants (Fig. 4J and K) or untreated *jaw*-D/*TCP10m*-*GR* and control plants (Fig. 4H, I, and L). These results are in agreement with the hypothesis that TCP4 and TCP10 share similar functions. To investigate how the effect of TCP10 on the apical meristem can be explained at the molecular level, this work determined potential direct target genes of *TCP10m-GR* by combining a DEX-induction assay with a microarray analysis. The resulting list of 89 genes that are differentially regulated between *jaw*-D/*TCP10m-GR* versus *jaw*-D treated with DEX is given in Supplementary Table S4. Surprisingly, this work did not identify in the potential *TCP10m-GR* target gene list any common genes or those that are known to be affected by TCP4 (Schommer *et al.*, 2008). However, in the previously performed experiments for TCP4, the focus was not on the detection of direct target genes, and steady-state expression differences between wild-type and *TCP4* overexpression lines were identified. Hence, overlap for particular affected biological processes only could be expected and so, considering this, the microarray data were analysed for overrepresentation of biological processes using BiNGO (Maere *et al.*, 2005) (Supplementary Table S5). Out of the genes that were included in this analysis, 12.6% were found to be involved in the response to auxin, whereas genome wide only 1% falls into this class. Although, after multiple-testing correction, the associated *P*-value is not significant, for the more general term ‘response to stimulus’ the overrepresentation is significant. The eight auxin response genes are listed in Table 2. Among them were five SAUR-like (SMALL AUXIN UP RNA-like) auxin-responsive protein family genes and TCP4 appeared to regulate expression of SAUR-like genes as well (Sarvepalli and Nath, 2011). Consequently, it can be assumed that auxin signalling is important for *jaw*-TCP functions in the SAM and is affected by both TCP4 and TCP10. The list of potential direct target genes was examined more closely and the *KNAT3* gene, encoding for a member of the class II knotted1-like homeobox gene family, was among the upregulated genes upon *TCP10m-GR* activation (Supplementary Table S4). This was particularly interesting as other *KNAT* genes have previously been shown to interact with *jaw*-TCPs in early leaf development (Li *et al.*, 2012).

**Table 2.** Identified TCP10 target genes that are proposed to be involved in the response to auxin stimuliGene names based on TAIR10 gene ontology classification.

AGI locus	Name
*AT1G29440*	*SAUR-like auxin-responsive protein family*
*AT1G29500*	*SAUR-like auxin-responsive protein family*
*AT1G29510*	*SMALL AUXIN UPREGULATED 68* (*SAUR68*)
*AT2G21220*	*SAUR-like auxin-responsive protein family*
*AT2G46690*	*SAUR-like auxin-responsive protein family*
*AT3G48360*	*BTB AND TAZ DOMAIN PROTEIN 2* (*bt2*)
*AT4G03400*	*DWARF IN LIGHT 2* (*DFL2*)
*AT4G38840*	*SAUR-like auxin-responsive protein family*

### Validation of redundancy predictions by a genetic approach

**Fig. 5.**
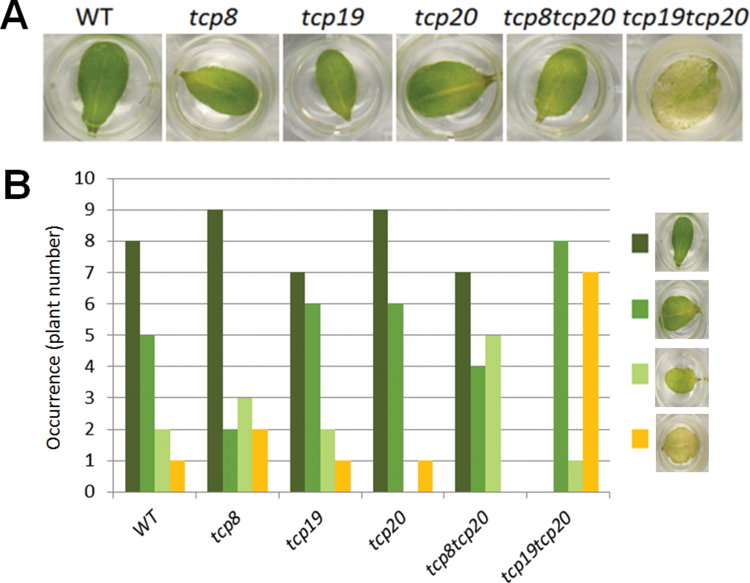
Phenotypic evidence for redundant functions between TCP19 and TCP20. The lines *tcp8*, *tcp19*, *tcp20*, and the double mutants *tcp8tcp20* and *tcp19tcp20* were subjected to a wound-induced senescence analysis together with the wild-type (WT) control. (A) A representative leaf for each analysed line after 4 days of incubation at room temperature in the dark. (B) In an assay involving 16 individual plants per line, *tcp19tcp20* leaves showed earlier senescence in two independent experiments. Leaves of the various plant lines were categorized into four different classes based on appearance. The differences between *tcp19tcp20* and the other lines are significant (*P* < 0.01, chi-squared test).

Ultimate proof for overlap in functions can be obtained by comparing single and double knockouts for particular combinations of *TCP* genes. Therefore, the bioinformatics analyses were further validated by crossing single T-DNA insertion lines for selected class I *TCP* pairs, followed by phenotypic analyses for the obtained single and double mutants. For this purpose, the pair *TCP20* and *TCP8* and the combination *TCP20* and *TCP19* were chosen. Whereas the pair TCP19–TCP20 ranked in the top 10 of both analyses for potential functional overlaps, the TCP8–TCP20 pair was not, despite TCP8 being as closely related to TCP20 in protein sequence as TCP19 (Fig. 1A). Recently, functions in determining leaf pavement cell sizes and in controlling the onset of senescence could be assigned to TCP20 (Danisman *et al.*, 2012). Therefore, a detailed senescence assay was performed to study possible overlap in functions for the indicated type I *TCP* gene pairs (Fig. 5A). As expected based on the bioinformatics predictions, the *tcp8tcp20* double mutant did not exhibit any accelerated senescence when subjected to the assay. Instead, the *tcp19tcp20* double mutant showed a greatly enhanced senescence phenotype, thus confirming that TCP19, but apparently not TCP8, shares function with TCP20 in the leaf senescence response (Fig. 5B). The senescence assay was analysed using a chi-squared test and the observed differences in the senescence frequencies of *tcp19tcp20* double mutants in comparison to wild type and the single mutants proved to be statistically significant with a *P*-value < 0.01. This confirms the hypothesis that using protein sequence information alone can be too limited for the identification of potential functionally redundant protein pairs and that a relatively simple bioinformatics analysis of available and easily obtainable data can increase the chance to detect functionally redundant proteins.

## Discussion

This study used an integrated bioinformatics approach to assess the *Arabidopsis* TCP transcription factor family to identify possible functional redundancies with a focus on the vegetative stage of development. Three data sources have been integrated into one output, namely protein sequences, RNA expression levels, and information about protein–protein interaction capacities. Subsequently, a ranking was obtained according to similarity in all datasets. For this purpose, the sum of ranks was calculated for each TCP pair, representing a score for functional redundancy potential.

### Groups of TCP transcription factors regulate different biological processes

TCP transcription factors have been identified to play roles in different biological processes, such as leaf development, axillary meristem outgrowth, and regulation of floral symmetry. In many of these cases, not a single *TCP* but a group of *TCP* genes is involved in the regulation of these processes (Martín-Trillo and Cubas, 2010). This is why this study applied a bioinformatics pipeline developed for the prediction of redundancy to the TCP family and focused on TCP pairs that potentially share roles in *Arabidopsis* leaf growth. This work found that known redundant gene pairs, such as *TCP4* and *TCP10*, were ranked high in the list of possibly redundant gene pairs. In order to obtain insight into the molecular mechanisms underlying the shared functions, a simple analysis was conducted to see if *TCP10* and *TCP4* indeed affect similar processes. Overexpression of a microRNA-resistant version of *TCP10* leads to developmental arrest in early development in the same way as it does in *TCP4m*-overexpressing plants (Palatnik *et al.*, 2003). Whereas the effects of *TCP4*, *TCP10*, and all other *jaw*-*TCP* genes on leaf development are well studied, this work took the opportunity to identify possible direct targets affected in early development. Here, genes for different biological processes were found to be enriched, including genes that respond to auxin stimuli such as five members of the *SAUR*-like auxin-responsive protein family. Evidence is accumulating that *SAUR* genes are associated with *jaw*-*TCP* genes in leaf development. When fused to a SRDX repressor domain, the class II TCP3 protein represses the expression of the two *SAUR* genes *At1g29460* and *At5g18020* (Koyama *et al.*, 2010). Accordingly, overexpression of *TCP3* and, amongst others, the SAUR gene *At1g29460* led to fused cotyledons and absence of SAMs, similar to the effect seen by *TCP10* overexpression in this analysis. This is in line with the observation that *TCP3–TCP10* score fourth and twelfth highest in the two individual rankings, respectively. Although the *SAUR* genes that are affected by the different TCP proteins are not the same, this work proposes that the interaction between *TCP* and *SAUR* genes, and hence the interaction between these particular *TCP* transcription factors and the auxin response pathway, is important for the maintenance of a functional SAM. Additionally, both *Jaw–TCP* genes and *SAUR* genes appeared to share functions in the control of leaf senescence (Schommer *et al.*, 2008; Hou *et al.*, 2013) providing additional evidence for a close relationship between these two gene families.

Another interesting target of TCP10 is *KNAT3*, a member of the class II *knotted1*-like homeobox gene family (Truernit and Haseloff, 2007). The expression of *KNAT3* is upregulated upon activation of *TCP10m-GR*. In a recent publication, Li *et al.*, (2012) showed that *jaw-*TCP proteins physically interact with the two transcription factors ASYMMETRIC LEAVES1 and 2 and repress the expression of the class I *knotted*-like homeobox genes *SHOOT MERISTEMLESS* (*STM*), *BREVIPEDICELLUS* (*BP*), *KNAT2*, and *KNAT6*. Future studies need to show if there is also a role for *KNAT3* in shoot development and if upregulation of this gene can lead to the phenotypes obtained upon overexpression of members from the *jaw-*TCP clade.

### Identifying functionally redundant class I TCP pairs

In contrast to the situation for class II *TCP* genes, the current knowledge concerning functions of class I *TCP* genes is limited. This bioinformatics analysis resulted in the identification of both known and unknown combinations of class I TCP transcription factors that possibly act redundantly. Importantly, integrating data that is already available or easily obtainable helped to exclude pairs of class I *TCP* genes that would otherwise be obvious choices for genetic studies when taking into account only sequence similarity. For example, the protein encoded by the *TCP20* gene is closely related to the TCP6 protein at sequence level. Without any gene expression analyses, however, this study would not have been able to exclude *TCP6* as a potential redundant gene, as it showed no expression in any of the expression datasets. Although a redundant function in a particular tissue not investigated cannot be ruled out, in the majority of tissues *TCP6* and *TCP20* are not concomitantly expressed. Similarly, TCP8, which is close to TCP20 in protein sequence, could be excluded due to different protein–protein interaction patterns and expression patterns for the corresponding genes. In line with these observations, a subsequent functional analysis based on the generation of single and double mutants did not reveal any obvious phenotypes pointing towards a joined function. On the other hand, the TCP19 and TCP20 proteins are quite distinct in sequence, but they appeared in the top 10 after the integrated bioinformatics analysis. In agreement with this, genetic analysis showed that double mutants resulted in earlier senescence when compared to single mutants, indicating that *TCP19* acts redundantly with the class I *TCP20* gene (Danisman *et al.*, 2012). Although this study cannot fully rule out the possibilities of *TCP20* being active in parallel pathways and the presence of a strong synergistic effect due to the double knockout, the performed analyses strongly support a redundant function for TCP19 and TCP20 in the senescence response. Herewith it could be shown that similarities in protein sequence alone can be limited in the search for redundant protein pairs and, preferably, should be complemented with further molecular data sets. Furthermore, proteins that do not group together in a phylogenetic tree but overlap in protein interaction and expression patterns may act redundantly in the plant. Nevertheless, the method failed to detect the known redundant *TCP14* and *TCP15* genes as top candidate pair. A recent functional analysis revealed redundant functions for these two class I *TCP* genes in regulating internode length and leaf development (Kieffer *et al.*, 2011). Although the two TCPs show similarity in protein–protein interactions, there is a strong difference in the overall *TCP14* and *TCP15* expression patterns (Kieffer *et al.*, 2011). The current method is based on the assumption that functionally redundant genes should have similar expression patterns and, hence, this type of examples will be missed. However, it also suggests that *TCP14* and *TCP15* share functions only partially and may fulfil different roles in the organs where they are not co-expressed. Furthermore, it is important to realize that only redundancies between pairs of TCPs were analysed. Nevertheless, indirect indications about potential multiple gene redundancies can be extracted from this pair-wise analysis. For example, the pairs TCP4–TCP10, TCP4–TCP3, and TCP10–TCP3 can be found at rank 4, 6 and 12, respectively (Supplementary Table S3), suggesting functional redundancy for these three TCP transcription factors. Indeed, overlap in function was shown for the encoding *TCP* genes in experimental studies (Palatnik *et al.*, 2003; Schommer *et al.*, 2008; Nag *et al.*, 2009). Comparing protein sequences, co-expression and protein–protein interactions for three or more proteins directly is possible, but more complex. However, attempts in this direction have been undertaken recently on a genome-wide scale (Chen *et al.*, 2010). If the predictions made by such an approach are verifiable using molecular genetic studies, remains to be seen. Furthermore, approaches that target a single family most likely have a higher chance to find redundant genes than genome-wide analyses, because of the feasibility to perform limited but focused experiments to complement important missing data points and the possibility to optimize the analysis for a specific purpose. The focus of this study was on vegetative development and, hence, a leaf developmental gene expression time series was selected as input for the redundancy prediction. For the study of possible functional redundancies of *TCP* genes regarding hormone signalling or the SAM, for example, it would be an option to use the available expression data from hormone treatment experiments (Goda *et al.*, 2008) and the high-resolution expression data of the SAM stem cell niche (Yadav *et al.*, 2009), respectively.

This work showed that class I TCPs interact in the regulation of senescence and that double mutants show accelerated senescence behaviour. An effect of class I TCPs on senescence was shown in another of this group’s studies (Danisman *et al.*, 2012). However, that study only looked at the role of TCP9 downstream of TCP20 and there are no data indicating that other class I TCPs apart from *TCP9* are targets of TCP20, which suggests that TCP9 may be a downstream conductor of TCP20 regulation, but may not be considered a functional redundant homologue of TCP20. TCP9 was excluded from the bioinformatics analyses because it showed no dimerization capacity in the Y2H experiments. Consequently, no rank is available for the pair TCP9–TCP20. Target gene analyses would clarify the question as to how far the functions of TCP9 and TCP20 overlap. The closest homologue of TCP9 in protein sequence is TCP19, for which overlapping functions with TCP20 was shown. Accordingly, the current work proposes that the genes *TCP9*, *TCP19*, and *TCP20* are all involved in the regulation of leaf senescence and that they either share functions equally or *TCP19* and *TCP20* are upstream of the downstream conductor *TCP9* (Danisman *et al.*, 2012).

In conclusion, large-scale data analysis was combined with molecular biology approaches to study functionally redundant pairs of the TCP transcription factor family within *Arabidopsis* leaf development. Although some known redundant TCP pairs were not detected, a novel protein pair (TCP19–TCP20) that affects leaf development was identified, showing the feasibility of this approach.

## Supplementary material

Supplementary data are available at *JXB* online.

Supplementary Fig. S1. Correlation between calculated expression-based distances for pairs of *TCP* genes.

Supplementary Table S1. AtGenExpress data on *TCP* expression used for this study.

Supplementary Table S2. Predictions made for pairs of TCP transcription factors based on AtGenExpress data.

Supplementary Table S3. Predictions made for pairs of TCP transcription factors based on own qRT-PCR data for leaves.

Supplementary Table S4. Potential TCP10 target gene list based on *TCP10m*-*GR* microarray experiment.

Supplementary Table S5. Over-represented gene ontology classes in the potential TCP10 target gene list.

Supplementary Table S6. Primers used in this study.

Supplementary Data
