# Supplementary material for: Analysis of functional redundancies within the Arabidopsis TCP transcription factor family
Source: J Exp Bot. 2013 Oct 15;64(18):5673–85. doi: 10.1093/jxb/ert337 (PMC3871820; doi:10.1093/jxb/ert337)
Supplement: Supplementary Data [file supp_64_18_5673__index.html]

Analysis of functional redundancies within the Arabidopsis TCP transcription factor family — Analysis of functional redundancies within the Arabidopsis TCP transcription factor family — Supplementary Data 

# Analysis of functional redundancies within the *Arabidopsis* TCP transcription factor family

## Supplementary Data

Data files

**Files in this Data Supplement:**

- Supplementary Data - Supplementary Data
- Supplementary Data - Supplementary Data
