# Supplementary material for: Analysis of functional redundancies within the Arabidopsis TCP transcription factor family
Source: J Exp Bot. 2013 Oct 15;64(18):5673–85. doi: 10.1093/jxb/ert337 (PMC3871820; doi:10.1093/jxb/ert337)
Supplement: Supplementary Data [file supp_ert337_jexbot104521_file002.pdf]

Analysis of functional redundancies within the *Arabidopsis* TCP transcription factor family

Selahattin Danisman, Aalt D.J. van Dijk, Andrea Bimbo, Froukje van der Wal, Lars Hennig, Stefan de Folter, Gerco C. Angenent, and Richard G.H. Immink

SUPPLEMENTARY DATA:

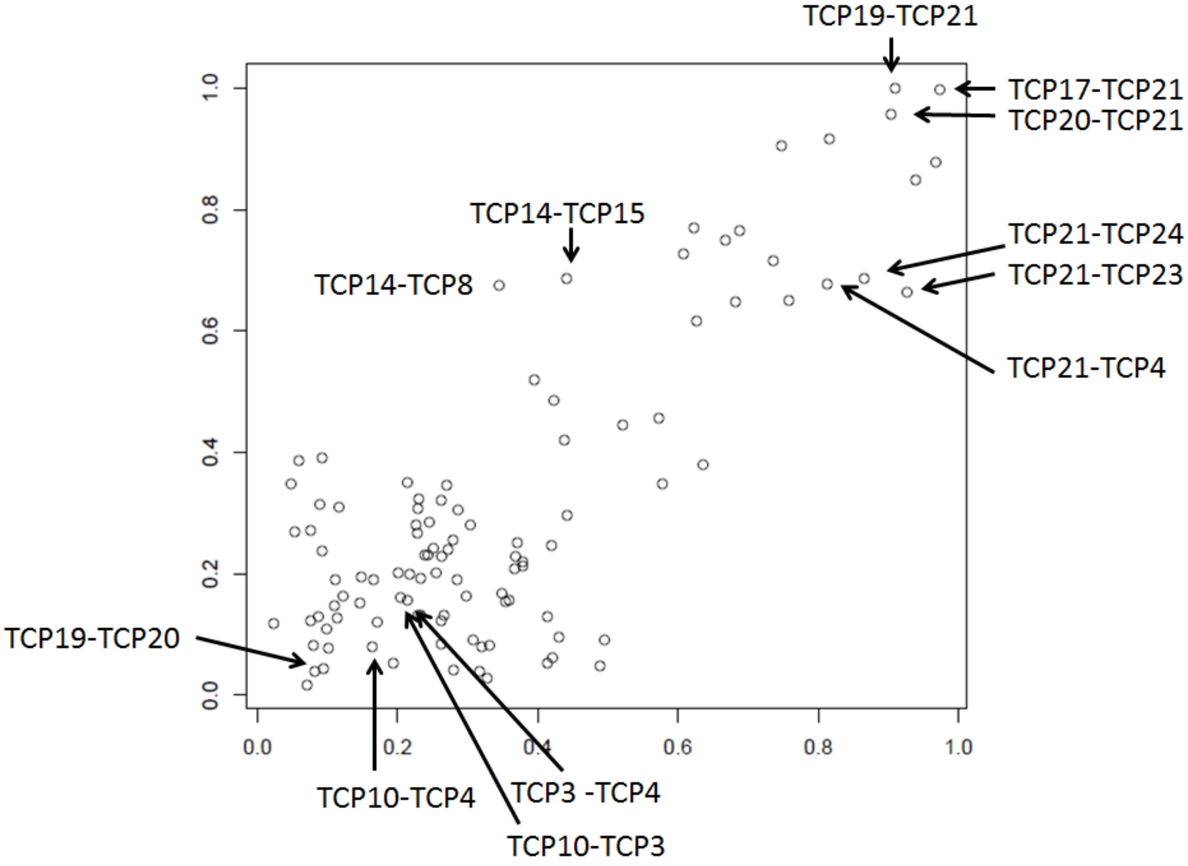

Figure S1: Correlation between calculated expression-based distances for pairs of *TCP* genes. X-axes: Distance scores for a leaf developmental qRT-PCR-based dataset (this study). Y-axis: Distance scores based on using the AtGenExpress dataset (Schmid et al, 2005). Labels indicate selected pairs with high or low expression-based distances (dissimilar or similar expression patterns, respectively), and selected pairs mentioned in the text.

References:

Schmid M, Davison TS, Henz SR, Pape UJ, Demar M, Vingron M, Scholkopf B, Weigel D, Lohmann JU (2005) A gene expression map of Arabidopsis thaliana development. *Nature* **37:** 501-506
